# DDX24 regulates the chemosensitivity of hepatocellular carcinoma to sorafenib via mediating the expression of SNORA18

**DOI:** 10.1080/15384047.2022.2135960

**Published:** 2022-10-30

**Authors:** Hairun Gan, Luting Li, Xinyan Hu, Jianxun Cai, Xiaojun Hu, Haopei Zhang, Ni Zhao, Xiwei Xu, Hui Guo, Pengfei Pang

**Affiliations:** a Center for Interventional Medicine, the Fifth Affiliated Hospital, Sun Yat-sen University, Zhuhai, Guangdong Province, China; b Guangdong Provincial Key Laboratory of Biomedical Imaging, the Fifth Affiliated Hospital, Sun Yat-sen University, Zhuhai, Guangdong Province, China; c Guangdong Provincial Engineering Research Center of Molecular Imaging, the Fifth Affiliated Hospital, Sun Yat-sen University, Zhuhai, Guangdong Province, China; d The Cancer Center of the Fifth Affiliated Hospital of Sun Yat-sen University, Zhuhai, Guangdong Province, China

**Keywords:** Hepatocellular carcinoma (HCC), sorafenib (SFN), DDX24, SNORA18, chemosensitivity

## Abstract

Sorafenib (SFN) is a multi-kinase inhibitor drug for the treatment of advanced hepatocellular carcinoma (HCC), but its limited efficacy is a major obstacle to the clinical outcomes of patients with HCC. We aimed to explore a novel molecular mechanism underlying the chemosensitivity of HCC to SFN, and to identify a promising therapeutic target for HCC treatment. In this study, bioinformatic analysis revealed that DDX24 was associated with poor survival in HCC cases, and significantly related to the pathways modulating tumor development. DDX24 regulated HCC cell proliferation and migration potentials. Moreover, reduction of DDX24 promoted the sorafenib-mediated inhibition of HCC cell growth and migration, the elevation of sorafenib-induced HCC cell apoptosis. DDX24 overexpression suppressed the inhibitory effect of SFN on cell proliferation and migration and reduced the apoptosis induced by SFN. Further, DDX24, combined with SFN treatment, presented a synergistic enhancement of the sensitivity of SFN to the growth and migration of HCC cells via AKT/ERK and the epithelial-mesenchymal transition (EMT) pathways, and that it modulated apoptosis via the caspase/PARP pathway. Mechanistically, SNORA18 served as a target gene for DDX24, regulating the chemosensitivity of sorafenib-treated HCC cells. Furthermore, SNORA18 knockdown or overexpression could partially reverse the inhibition or elevation of cell viability, colony formation and migration induced by DDX24 in sorafenib-treated HCC cells, respectively. Collectively, our results suggest that DDX24 regulates the chemosensitivity of HCC to SFN by mediating the expression of SNORA18, which may act as an effective therapeutic target for improving SFN efficiency in HCC treatment.

## Introduction

Hepatocellular carcinoma (HCC) is the sixth most common cancer worldwide and ranks third as a leading cause of cancer-related death, with a mortality rate of 82%.^1^ HCC is often diagnosed late due to the asymptomatic nature of this disease in the early stage, and it is not feasible to achieve curative treatment in patients with advanced HCC.^2^ The asymptomatic onset, rapid tumor progression, therapy resistance, cancer recurrence and distant metastasis are the principal causes accounting for the poor prognosis of HCC, resulting in a severe burden for healthcare systems.^2,3^

Sorafenib (SFN), a tyrosine kinase inhibitor (TKI), is the first-line FDA-approved drug for the treatment of unresectable advanced HCC, providing a 44% improved overall survival (OS) rate in patients.^4^ Despite the encouraging efficacy of SFN, its effectiveness is mainly compromised by a decline in sensitivity, and acquired chemoresistance.^5^ The specific mechanisms of the distinct sensitivities of patients with HCC administered SFN are complicated. This is ascribed to the complex interaction of multiple factors, including the activation or dysregulation of various signaling pathways, hypoxia-inducible responses, enrichment of the liver cancer stem cells (LCSC) and mitophagy of reactive oxygen species.^6,7^ Therefore, enhancing SFN sensitivity in HCC treatment is of great value for improving the OS of patients with HCC, and elucidating the molecular regulation underlying drug sensitivity is vital.

DEAD-box proteins are the largest family of RNA helicases with ATPase activity and function in numerous aspects of eukaryotic RNA metabolism.^8^ DDX24 is an important member of the DEAD-box RNA helicases carrying a conserved Asp-Glu-Ala-Asp (D-E-A-D) motif.^9^ Based on our recent findings, a mutated *DDX24* gene could cause vascular malformations,^10^ and previous studies have revealed that DDX24 is upregulated in multiple human cancer cells, inhibiting cell growth by inducing cell cycle arrest and senescence in osteosarcoma cells.^11^ Moreover, DDX24 also regulates the proliferation of colon cancer and gastric cancer cells.^12^ Most published research associated DDX24 strongly with the development of carcinoma, however, the specific role of DDX24 in HCC is still obscure.

Small nucleolar RNA (snoRNA) is a massive subgroup of non-coding RNA (ncRNA) with a length of 60–300 nucleotides predominately found in the eukaryotic nucleolus.^13^ Based on the structural elements of snoRNAs, they are categorized into three types containing the C/D box, ribonuclease 7–2/MRP and H/ACA box snoRNAs, which directly bind to their substrate via base pairing for inducing 2′O-methylation and pseudoacylation, respectively.^14^ Since the snoRNAs are crucial regulatory factors responsible for post-transcriptional modification of ribosomal RNAs (rRNAs), spliceosomal RNAs (snRNAs) and transfer RNAs (tRNAs), they can modulate various physiological and pathological processes, such as cell proliferation, differentiation and angiogenesis.^15^ From recent studies, an elevated snoRNAs expression could significantly induce oncogenesis of different carcinoma due to a disordered synthesis of ribosomes. Additionally, snoRNAs have been proven to be closely related to the prognosis of patients with HCC and a potential molecular therapeutic target in HCC.^16^ Small nucleolar RNA H/ACA box 18 (SNORA18) transcribed from the chromosome 11q21 genomic region was identified as a regulator of tumor growth in pancreatic cancers.^17,18^ Currently, evidence assessing the particular effect of SNORA18 in the progression of HCC, and mediating the chemosensitivity of SFN, remains elusive.

In this study, we examined the functional role of DDX24 on the development of HCC and on the modulation of the sensitivity to SFN in HCC treatment. Furthermore, we elucidated a potential mechanism by which DDX24 regulated SFN sensitivity in HCC therapy by mediating the expression of its target SNORA18.

## Results

### High DDX24 expression is associated with poor survival in HCC

**Figure 1.**
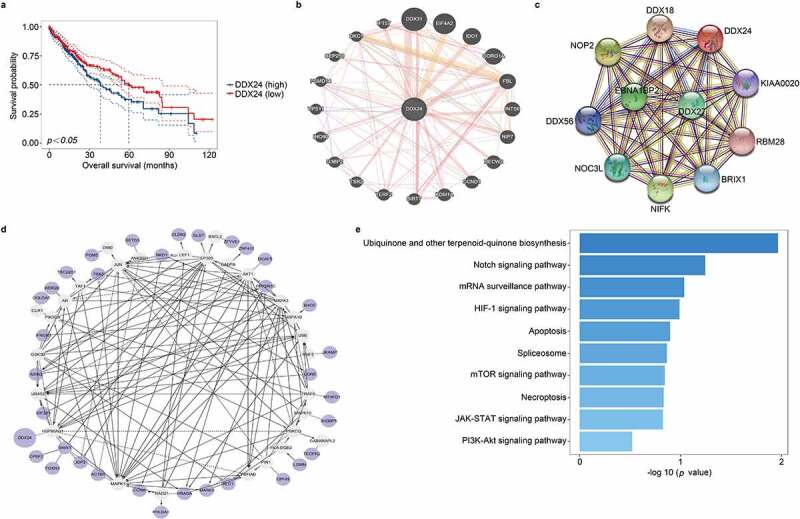
Overexpression of DDX24 correlates with poor survival, and the functional enrichment analysis of DDX24 in HCC. (a) Kaplan-Meier overall survival analysis of liver cancer tissues with high (blue line) or low (red line) DDX24 expression based on the data obtained from TCGA dataset. (b) Network map showed the relationships between DDX24 and neighboring genes using GeneMANIA tool. (c) The PPI network of DDX24 was conducted using STRING tool. (d) Interaction network map on the top 50 co-expressed genes of DDX24 (*P < .05*) was conducted using Cytoscape tool. The purple circles were interacted genes, the white circles were un-interacted genes. (e) KEGG pathway enrichment analysis on the top 18 co-expressed genes of DDX24 (|Spearman correlation coefficient| >0.4 and *p < .01*) in liver cancer samples obtained from TCGA dataset.

To explore the specific function of DDX24 in HCC, we first analyzed 371 liver cancer samples and 50 adjacent samples from The Cancer Genome Atlas (TCGA) database. We divided patients with liver cancer from the TCGA dataset into two groups based on their DDX24 expression. A Kaplan-Meier analysis indicated that the DDX24 level was strongly correlated with patient’s overall survival time (*p < .05*, Figure 1a). Subsequently, we explored the potential biological pathways associated with DDX24 to evaluate its molecular mechanism in HCC. The gene network map using the GeneMANIA tool demonstrated that DDX24 was surrounded by 20 nodes representing genes with close correlations (Figure 1b), and the protein-protein interaction (PPI) network using the STRING tool revealed that DDX24 was connected with 10 proteins (Figure 1c). We found DDX24 was correlated with DKC1, CEP250, PSMD14, INO80, G3BP2, SIRT7, KDM1A, CCND1, FBL, IDO1 and DDX27, functioning as an oncogene in the development of HCC.^19–21^ Furthermore, we identified the top 50 co-expressed genes of DDX24 in liver cancer samples from the TCGA database and performed an interaction network analysis via the Reactome Pathway (*p < .05*, Figure 1d). Next, the top 18 co-expressed genes (|Spearman correlation coefficient| >0.4 and *p < .01*, Supplementary Table 1) were subjected to the Kyoto Encyclopedia of Genes and Genomes (KEGG) analysis. A KEGG pathway enrichment analysis revealed that DDX24 was likely involved in crucial biological signaling relating to promotion of HCC progression, including PI3K-Akt-mTOR, apoptosis, JAK-STAT, Notch and the Necroptosis signaling pathways (Figure 1e).^22,23^ In summary, a high expression of DDX24 is significantly related to patient overall survival, and DDX24 may be involved in the pathways regulating HCC development.

### DDX24 regulates proliferation and migration of HCC cells

**Figure 2.**
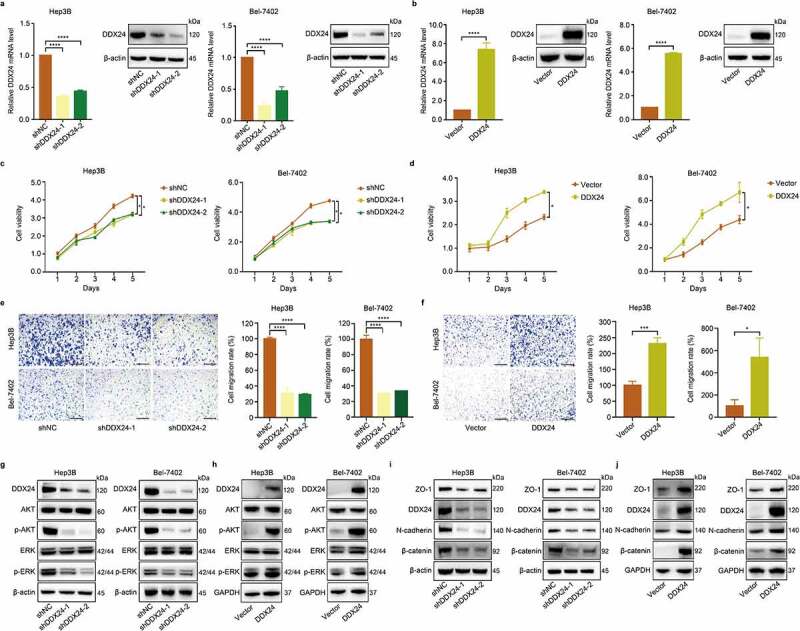
DDX24 regulates proliferation and migration of HCC cells. (a, b) Efficacy of DDX24 knockdown (a) or overexpression (b) in Hep3B and Bel-7402 cells was analyzed by the RT-qPCR and western blot analysis. (c, d) Cell viability was detected by CCK-8 assays after Hep3B and Bel-7402 cells transfecting with DDX24-specific shRNAs (c) or DDX24 plasmid (d). (e, f) Cell migration rates of Hep3B and Bel-7402 cells transfected with shRNAs (e) or DDX24 plasmid (f) were measured by trans-well assays, and representative qualification was shown in the right panel. Scale bar = 100 μM. (g-j) Western blot analysis of DDX24, AKT, p-AKT, ERK, p-ERK, ZO-1, N-cadherin and β-catenin expression after DDX24 knockdown or overexpression in Hep3B and Bel-7402 cell lines. β-actin or GAPDH was used as the loading control. The results were shown as means ± SD, *p < .05, ** p < .01, *** p < .001, *** p < .0001; n. s., not significant.

As the KEGG pathway enrichment analysis showed that DDX24 was correlated with HCC development, we, therefore, reduced and elevated the expression of DDX24 in Hep3B and Bel-7402 cells to further investigate the effect of DDX24 on HCC tumorigenesis. Real-time quantitative polymerase chain reaction (RT-qPCR) and western blot analysis were used to confirm the alteration of DDX24 levels (Figure 2a,). We found that DDX24 knockdown and overexpression significantly inhibited and promoted the cell proliferation and migration potentials of HCC cells, respectively (Figure 2c-f). Consistent with these results, the suppression of DDX24 in Hep3B and Bel-7402 cells also remarkably decreased the phosphorylation of AKT and ERK, and the expression of epithelial-mesenchymal transition (EMT) associated proteins ZO-1, N-cadherin and β-catenin (Figure 2g,). However, DDX24 overexpression had a converse effect on protein levels (Figure 2h,). These data indicate that DDX24 regulates the proliferation and migration of HCC cells *in vitro*.

### DDX24 regulates sorafenib-mediated inhibition of proliferation in HCC cells

**Figure 3.**
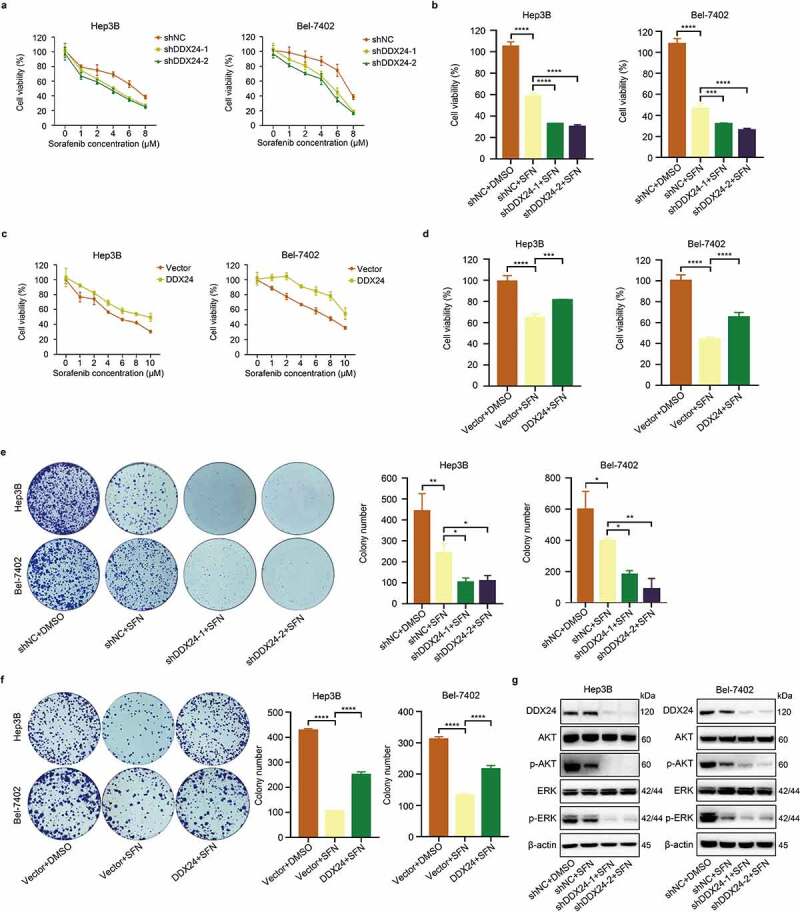
DDX24 regulates sorafenib-mediated inhibition of proliferation in HCC cells. (a, c) Cytotoxic effect of sorafenib (SFN) at the indicated doses on Hep3B and Bel-7402 cells transfected with DDX24-specific shRNAs (a) or DDX24 plasmid (c) was determined by CCK-8 assays after 48 hr, and IC50 of the cell lines exposed to SFN was analyzed. (b, d) Cell viability of Hep3B and Bel-7402 cells after transfecting with shRNAs (b) or DDX24 plasmid (d) followed by the treatment with SFN was detected by CCK-8 assays. (e, f) Colony formation assays of DDX24 knockdown (e) or overexpression (f) Hep3B and Bel-7402 cells treated with DMSO or SFN for 14 days, and representative qualification was shown in the right panel. (g) Expression of DDX24, AKT, p-AKT, ERK, p-ERK was tested by the western blot analysis. β-actin was used as the loading control. The results were shown as means ± SD, *p < .05, ** p < .01, *** p < .001, *** p < .0001; n. s., not significant.

Sorafenib (SFN) is a multi-kinase inhibitor approved as the standard first-line treatment for advanced HCC cases. However, the decline in sensitivity to SFN limits its efficacy in improving survival time.^4^ Recent studies have reported that the AKT/ERK pathway is involved in HCC sensitivity to SFN,^5,6^ and our data demonstrated that DDX24 could regulate the expression of phospho-AKT and phospho-ERK. We speculated that DDX24 might regulate the sensitivity of HCC cells to SFN treatment. To verify this hypothesis, HCC cells with DDX24 silencing were administrated to a medium containing different concentrations of SFN. We found that reduction of DDX24 increased the sensitivity of HCC cells to SFN via cell viability analysis, and the IC50 values of SFN in DDX24 knockdown Hep3B and Bel-7402 cells were lower (Hep3B shDDX24-1: 4.23 ± 0.19 μmol/l, Hep3B shDDX24-2: 4.82 ± 0.18 μmol/l, Bel-7402 shDDX24-1: 4.93 ± 0.50 μmol/l, Bel-7402 shDDX24-2: 4.08 ± 0.40 μmol/l) in contrast to the corresponding control cells (Hep3B shNC: 5.58 ± 0.08 μmol/l, Bel-7402 shNC: 7.04 ± 0.18 μmol/l) (Figure 3a). However, the inhibitory effect of SFN on DDX24 overexpressing cells was weaker than that of the vector group in cell viability (Figure 3c). As shown in Figure 3b, CCK-8 assays showed that downregulation of DDX24 dramatically increased the suppression of SFN on Hep3B and Bel-7402 proliferation. Moreover, we also found that the inhibitory effect of SFN on DDX24 knockdown cells was stronger than a nonspecific shRNA control using colony formation assays (Figure 3e). Meanwhile, overexpression of DDX24 reduced the inhibitory effect of SFN on cell proliferation and colony formation (Figure 3d,). We next measured the pivotal protein expression of the AKT/ERK signaling pathway. The results revealed that SFN treatment decreased the phosphorylation of AKT and ERK, but had no effect on DDX24, total AKT and total ERK expression. Additionally, silencing DDX24 was shown to further enhance the SFN suppression of phospho-AKT and phosphor-ERK proteins (Figure 3g). These findings suggest that DDX24 regulates sorafenib-mediated inhibition of HCC cell proliferation via the AKT/ERK pathway *in vitro*.

### DDX24 regulates sorafenib-induced apoptosis in HCC cells

**Figure 4.**
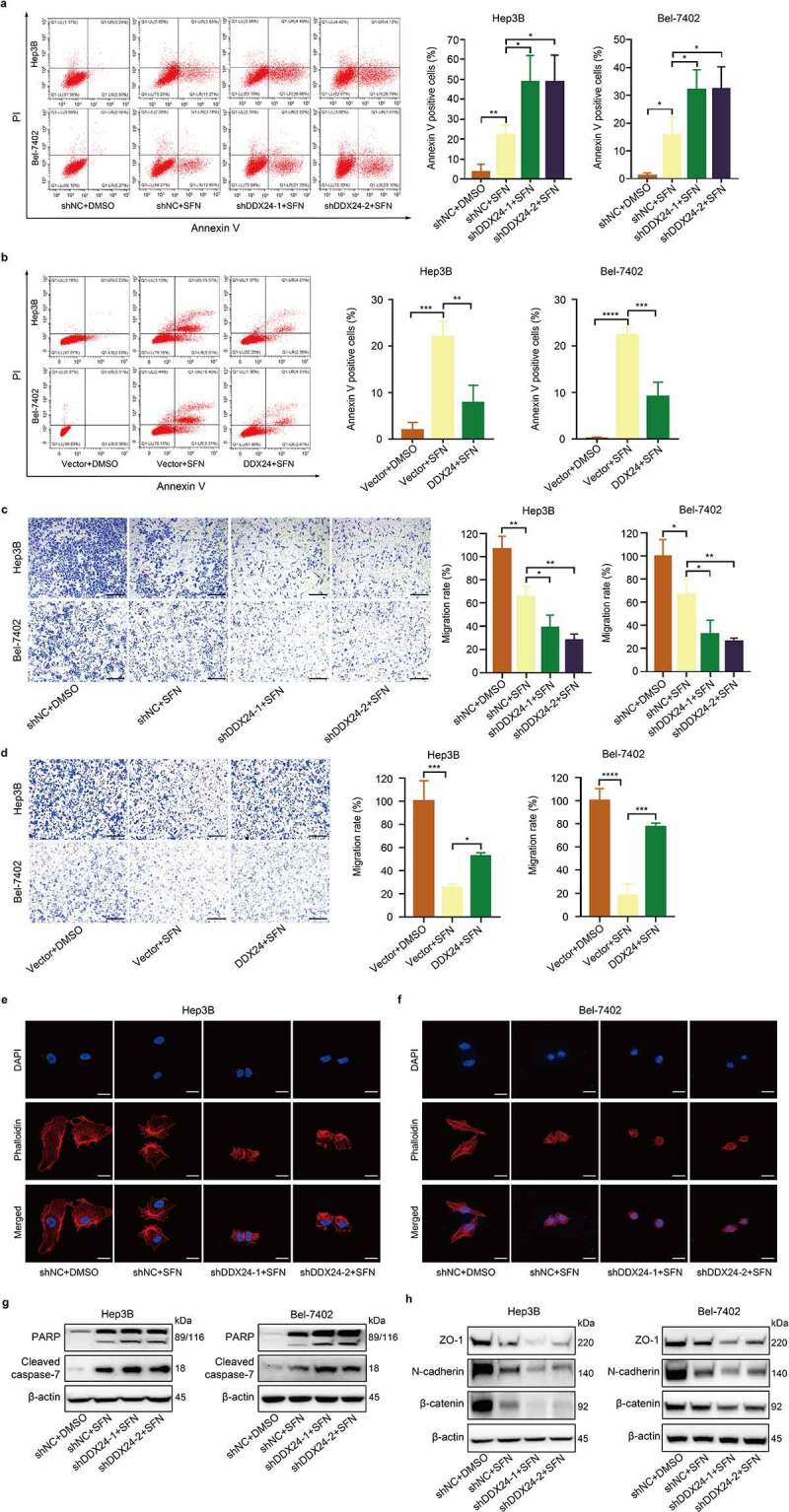
DDX24 regulates sorafenib-induced apoptosis and sorafenib-mediated inhibition of migration in HCC cells. (a, b) Apoptosis rates of Hep3B and Bel-7402 cells transfected with DDX24-specific shRNAs (a) or DDX24 plasmid (b) followed by the treatment with SFN for 48 hr were determined by flow cytometry assays, and representative qualification was shown in the right panel. (c, d) Cell migration rates of DDX24 knockdown (c) or overexpression (d) Hep3B and Bel-7402 cells treated with DMSO or SFN for 48 hr were detected by trans-well assays, and representative qualification was shown in the right panel. Scale bar = 100 μM. (e, f) Representative immunofluorescent images of phalloidin (red) in Hep3B (e) and Bel-7402 (f) cells transfected with shNC, shDDX24-1 and shDDX24-2 followed by the treatment with SFN. Blue, nucleus. Scale bar = 5 μM. (g, h) Expression of PARP, cleaved caspase-7, ZO-1, N-cadherin and β-catenin proteins was evaluated by the western blot analysis. β-actin was used as the loading control. The results were shown as means ± SD, *p < .05, ** p < .01, *** p < .001, *** p < .0001; n. s., not significant.

We next explored whether apoptosis mediated the anti-proliferation activity induced by DDX24 and SFN treatment in HCC cells. The flow cytometry assays using Annexin V-FITC/PI double staining revealed that the combined treatment of DDX24 knockdown and SFN exerted a synergistic effect on elevating cell apoptosis in Hep3B and Bel-7402 cells, compared with SFN treatment alone (Figure 4a). Conversely, we found DDX24 overexpression decreased the cell apoptosis induced by SFN (Figure 4b). Additionally, we measured the downstream apoptosis-related protein expression using western blot analysis. The results showed that SFN administration increased the expression of cleaved-PARP and cleaved caspase-7, and this trend was further promoted following DDX24 inhibition (Figure 4g). Together, these data show that DDX24 regulates sorafenib-induced apoptosis in HCC cells via the caspase/PARP pathway *in vitro*.

### DDX24 regulates sorafenib-mediated inhibition of migration in HCC cells

Metastasis is a crucial determinant of cancer-related prognosis and OS time, and mounting evidence has suggested that the EMT signaling pathway plays a significant role in HCC metastasis and its sensitivity to SFN.^7^ As our results demonstrated that DDX24 could modulate the expression of EMT related proteins, we aimed to detect whether DDX24 combined with SFN treatment performed a synergistic inhibition on HCC cell migration. In our study, migration assays indicated that the inhibition of cell migration rate caused by SFN was remarkably increased by DDX24 knockdown in Hep3B and Bel-7402 cells (Figure 4c). However, the inhibition of migration mediated by SFN could be reversed via DDX24 overexpression (Figure 4d). We next investigated the cytoskeleton using IF assays and found that SFN treatment reduced the number of spike-like filopodia at the edges of HCC cells, while this phenomenon was also more prominent following DDX24 silencing (Figure 4e,). Furthermore, western blot analysis revealed that the expression of EMT associated proteins ZO-1, N-cadherin and β-catenin, was suppressed in Hep3B and Bel-7402 cells treated with SFN, and that this was more dramatic in DDX24 knockdown cells (Figure 4h). Therefore, we conclude that DDX24 regulates the sorafenib-mediated inhibition of HCC cell migration via the EMT pathway *in vitro*.

### DDX24 knockdown elevates sorafenib-mediated inhibition of HCC growth in vivo

**Figure 5.**
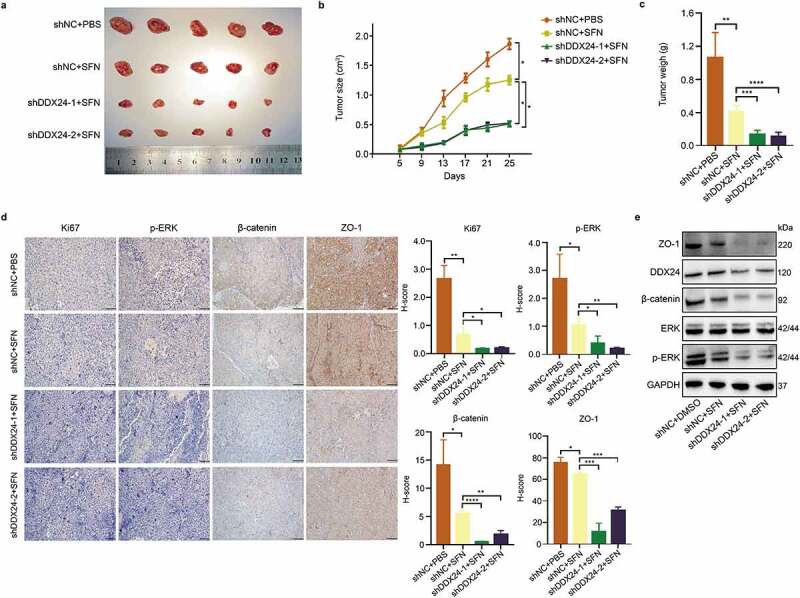
DDX24 knockdown elevates sorafenib-mediated inhibition of HCC growth *in vivo*. (a-c) Representative images of xenograft tumors derived from BALB/c nude mice (a) subcutaneously injected with DDX24 knockdown and negative control Hep3B stable cells, and administration with PBS or SFN (60 mg/kg) orally once daily. The tumor volume (b) and weight (c) were measured (N = 5). (d) Representative IHC staining of Ki67, p-ERK, β-catenin and ZO-1 from tumor sections, and representative qualification was shown in the right panel. Scale bar = 100 μM. (e) The expression of DDX24, ERK, p-ERK, β-catenin and ZO-1 in tumor xenografts was tested by western blot analysis. GAPDH was used as the loading control. The results were shown as means ± SD, *p < .05, ** p < .01, *** p < .001, *** p < .0001; n. s., not significant.

To investigate whether DDX24 regulated the anti-tumor effect of SFN against HCC growth *in vivo*, we used a subcutaneous mouse HCC xenograft model. Hep3B cells showing stable DDX24-shRNAs or control shRNA were injected subcutaneously into BALB/c nude mice aged four to six weeks. Tumor-bearing mice were administered with vehicle or SFN orally, once daily. All the mice tolerated the treatment well without observable signs of toxicity and had stable body weights throughout the study. As shown in Figure 5a-c, the *in vivo* anti-tumor assays revealed that the size and weight of xenografts in the control shRNA group receiving SFN treatment were decreased in contrast to the control shRNA group receiving PBS. Furthermore, a significant inhibitory effect on the tumor size and weight was exhibited in the DDX24-shRNAs group, compared to the control shRNA group receiving SFN. Consistent with the *in vivo* findings, immunohistochemistry (IHC) assays of xenografts generated from DDX24-shRNAs+SFN tumor tissues demonstrated an obvious reduction in Ki67, p-EKT, β-catenin and ZO-1 levels (Figure 5d). The western blot analysis also showed a declined expression of p-EKT, β-catenin and ZO-1 (Figure 5e). Collectively, these results confirm that DDX24 knockdown regulates the sorafenib-mediated inhibition of HCC growth *in vivo*.

### DDX24 regulates chemosensitivity of HCC cells to sorafenib via SNORA18 signaling

**Figure 6.**
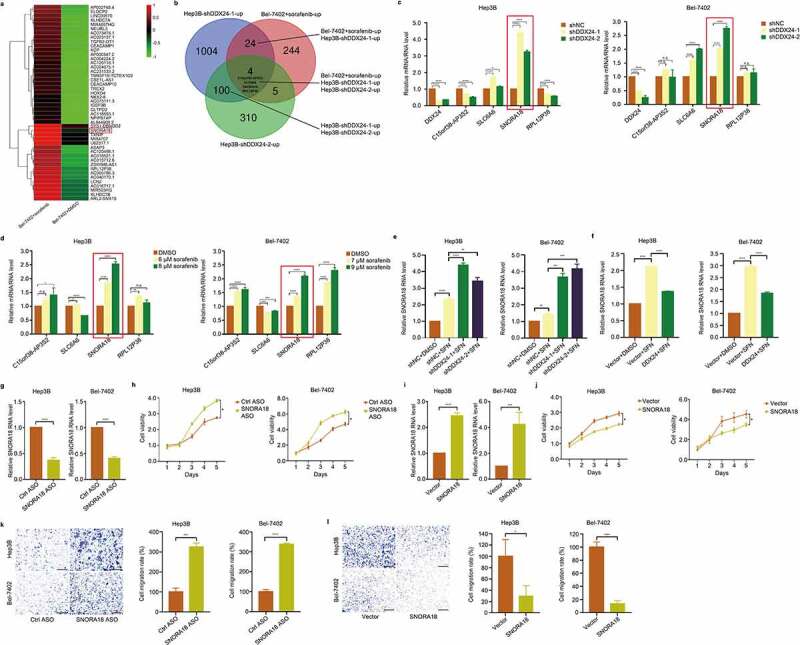
DDX24 regulates chemosensitivity of HCC cells to sorafenib via SNORA18 signaling. (a) Heat map from global comparative transcriptome analysis. Bel-7402 cells were treated with SFN at the indicated dose, and total RNA was extracted for analysis after 48 hr. (b) Results of RNA-seq on DDX24 knockdown Hep3B cells and Bel-7402 cells administrated with SFN were crosslinked to seek differentially regulated genes (log_2_FC ≥1.5, *p < .01*). Venn diagram showed a small subset candidates (4 simultaneous upregulated genes). (c, d) RT-qPCR was used to detect the relative RNA level of SNORA18 in Hep3B and Bel-7402 cells transfected with shNC, shDDX24-1 and shDDX24-2 (c), and exposed to SFN at the indicated doses (d). (e, f) Relative RNA level of SNORA18 in DDX24 knockdown (e) or overexpression (f) Hep3B and Bel-7402 cells treated with DMSO or SFN was determined by RT-qPCR. (g, i) Efficacy of SNORA18 knockdown (g) or overexpression (i) in Hep3B and Bel-7402 cells transfected with antisense oligonucleotide (ASO) or SNORA18 plasmid was analyzed by the RT-qPCR analysis. (h, j) Cell viability was detected by CCK-8 assays after Hep3B and Bel-7402 cells transfecting with SNORA18-specific ASO (h) or SNORA18 plasmid (j). (k, l) Cell migration rates of SNORA18 knockdown (k) or overexpression (l) Hep3B and Bel-7402 cells were measured by trans-well assays, and representative qualification was shown in the right panel. Scale bar = 100 μM. The results were shown as means ± SD, *p < .05, ** p < .01, *** p < .001, *** p < .0001; n. s., not significant.

**Figure 7.**
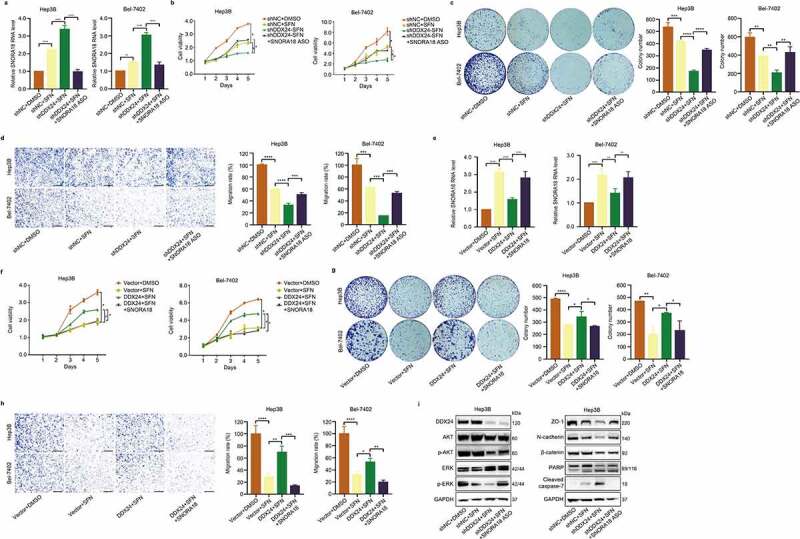
SNORA18 knockdown or overexpression reverts the chemosensitivity alteration induced by DDX24 in sorafenib-treated HCC cells. (a) RT-qPCR was used to detect the relative RNA level of SNORA18 in Hep3B and Bel-7402 cells transfected with shNC, shDDX24 and shDDX24+ SNORA18 ASO followed by the treatment with DMSO or SFN. (b-d) The shNC HCC cells exposed to DMSO and shNC, shDDX24, shDDX24+ SNORA18 ASO HCC cells exposed to SFN were subjected to cell viability (b), colony formation (c) and cell migration assays (d). Scale bar = 100 μM. (e-h) The vector HCC cells exposed to DMSO and vector, DDX24, DDX24+ SNORA18 HCC cells exposed to SFN were subjected to RT-qPCR (e), cell viability (f), colony formation (g) and cell migration assays (h). Scale bar = 100 μM. (i) Western blot analysis of DDX24, AKT, p-AKT, ERK, p-ERK, ZO-1, N-cadherin, β-catenin, PARP and cleaved caspase-7 expression after SNORA18 knockdown in Hep3B cells treated with DDX24-specific shRNA and SFN. GAPDH was used as the loading control. The results were shown as means ± SD, *p < .05, ** p < .01, *** p < .001, *** p < .0001; n. s., not significant.

To explore the underlying mechanism of DDX24 in regulating the chemosensitivity of HCC cells exposed to SFN, we performed RNA-seq comparing HCC cells treated with SFN to controls in order to determine aberrantly-expressed downstream genes. After the screening of differentially expressed RNAs by fold change (FC) filtering (log_2_FC ≥3.0) and student’s *t* testing (*p < .01*), there were 46 upregulated RNAs in Bel-7402 cells administrated with SFN versus the controls (Figure 6a). We then applied the result of RNA-seq on DDX24 knockdown Hep3B cells and a negative control from the online Gene Expression Omnibus (GEO) database (GEO Submission: GSE145635). As shown in Figure 6b, C15orf38-AP3S2, SLC6A6, SNORA18 and RPL12P38 emerged as candidates as they were both upregulated in HCC cells with SFN treatment and DDX24 knockdown. Furthermore, RT-qPCR analysis confirmed that SNORA18 was significantly increased when HCC cells were treated with SFN, and in HCC cells after DDX24 silencing (Figure 6c,). In addition, the expression of SNORA18 was remarkably elevated or reduced in DDX24 knockdown combined with SFN, or overexpression combined with SFN, respectively, in contrast to the group treated with SFN alone (Figure 6e,). We next sought to investigate the function of SNORA18 in HCC cells, and RT-qPCR was performed to measure the reduction and elevation of SNORA18 levels (Figure 6g,). The findings revealed that downregulation or upregulation of SNORA18 enhanced or suppressed the cell proliferation and migration abilities of Hep3B and Bel-7402 cells, respectively (Figure 6h, 6-l). We also found that the inhibition of cell viability, colony formation and migration induced by silencing DDX24 and SFN treatment was partially reversed through SNORA18 knockdown (Figure 7a-d). However, SNORA18 overexpression could partially reverse the DDX24-induced inhibitory effect of SFN on cell viability, colony formation and migration (Figure 7e-h). Moreover, reduction of SNORA18 in Hep3B cells treated with DDX24-specific shRNA and SFN could increase the expression of phosphorylation of AKT and ERK, ZO-1, N-cadherin and β-catenin, while decreasing the expression of cleaved-PARP and cleaved caspase-7 (Figure 7i). These findings indicate that DDX24 modulates the chemosensitivity of HCC cells to SFN via the SNORA18 pathway.

## Discussion

The most common malignant solid tumor of the liver, HCC accounts for 85% of all primary liver carcinoma cases.^1^ SFN is the first FDA approved multi-kinase inhibitor drug for the treatment of advanced HCC. However, the declined sensitivity and developed accompanying extension of the medication time, limit its improvement of the clinical outcomes of patients with HCC.^24^ Hence, it is urgent to explore novel therapeutic strategies to enhance the sensitivity of SFN in HCC treatment.

In this study, we found that DDX24 acted as an oncogene in HCC development via bioinformatic analysis, and DDX24 regulated HCC cell proliferation and migration by mediating the phosphorylation of AKT and ERK, and EMT protein levels. As SFN contributes mainly to the blockade of PI3K/AKT, MEK/ERK and the EMT signaling pathways,^25,26^ we further confirmed that a combination of DDX24 knockdown and SFN administration exerted a synergistic effect on suppressing HCC growth through modulating the AKT/ERK pathway *in vitro* and *in vivo*. Meanwhile, the results also revealed that downregulation or upregulation of DDX24 elevated or suppressed sorafenib-mediated inhibition of migration ability in HCC cells via the EMT pathway, respectively. Previous studies reported that SFN could induce caspase-mediated apoptosis in several human cancers.^26,27^ Our data showed that silencing DDX24 combined with SFN treatment presented a synergistic enhancement on HCC cell apoptosis through the caspase/PARP pathway. However, overexpression of DDX24 reduced the cell apoptosis induced by SFN treatment. To the best of our knowledge, this is the first study to systematically investigate the possible association between DDX24 and SFN treatment of HCC.

DDX24 is vital in plenty of cellular processes, for instance, DDX24 is implicated in the ribosome biogenesis.^28^ The defect of ribosomal maturation and function can result in the dysregulation of essential procedures, which transforms diseased and normal cells into cancer cells.^29^ Our previous research revealed that *DDX24* mutations were pathogenic factors of vascular deformities and played a significant role in the migration ability of endothelial cells.^10^ Based on published reports, DDX24 functions as a modulator of the p300-p53 axis by suppressing the p300-mediated acetylation of p53, which promotes the proliferation of osteosarcoma cells and lung carcinoma cells.^11^ Additionally, other studies revealed that a perturbation of DDX24 inhibited colon cancer and gastric cancer growth.^12^ Collectively, DDX24 functions as a new hallmark and therapeutic target for carcinoma, and our results, consistent with other published research, suggest that DDX24 can modulate the proliferation and migration potentials of HCC cells. Moreover, our findings also indicate that DDX24 may serve as a novel regulator of SFN sensitivity in HCC treatment.

Furthermore, understanding the specific molecular modulation on chemosensitivity of HCC to SFN can help identify potential targets and develop more effective therapeutic strategies against HCC. Mechanistically, we uncovered that DDX24 regulated the chemosensitivity of HCC cells to SFN by a small nucleolar RNA H/ACA box 18 (SNORA18) dependent pathway. Small nucleolar RNA (snoRNA) is a critical modulator participating primarily in the processing, folding and modification of pre-ribosomal RNAs (pre-rRNAs).^29^ This implies that its function may be correlated with the DDX24-mediated biogenesis of ribosomes. Recently, there has been an accumulation of interest in illustrating the malfunctioning roles of snoRNAs on the molecular mechanism of HCC development. For instance, SNORD76 enhanced the invasion of HCC cells through elevating the EMT pathway,^16^ while the snoU2_19 abrogation inhibited the HCC cell proliferation via Wnt/β-catenin signaling.^30^ Other reports have revealed that ACA11 knockdown represses the proliferation, migration and invasion of HCC cells,^31^ and SNORD52 highly expressed in HCC, could promote the development of HCC cells.^32^ However, the complicated relationship between snoRNAs and HCC is not clearly known, and there are few studies identifying the efficacy of snoRNAs on SFN sensitivity.

SNORA18 belonging to the H/ACA box snoRNAs is located at chromosome 11q21 within an intron of the small nucleolar RNA host gene TAF1D, which participates in the package of preinitiation complex (PIC) during RNA polymerase I dependent transcription.^33^ In a recent research, SNORA18 regulated the cell invasion and tumor metastasis of pancreatic cancers via binding to the KHSRP protein.^17^ Another study indicated that SNORA18, packed in intracellular vesicular endosomes, could be used as a diagnostic marker for differentiating patients with pancreatic dual adenocarcinoma (PDCA).^18^ All these reported findings suggest a possible role of SNORA18 in oncogenesis. The results of our study indicate that the combined treatment of DDX24 knockdown and SFN administration cause a synergistic increase in SNORA18 expression. The expression of SNORA18 was reduced in DDX24 overexpression HCC cells incubated with SFN, in contrast to the group treated with SFN alone. Moreover, silencing SNORA18 partially reversed the reduction of cell proliferation, colony formation and migration abilities induced by DDX24 knockdown and SFN treatment. Based on our data, we also found that SNORA18 knockdown could reverse the alteration of biological function in HCC cells treated with DDX24-specific shRNA and SFN by regulating the AKT/ERK, EMT and caspase/PARP pathways. As DDX24 and snoRNAs can both mediate ribosome biogenesis, we speculate that the DDX24/SNORA18 axis modulates the sensitivity of HCC cells administered with SFN by affecting the ribosomal synthesis. Details about the specific mechanism of this hypothesis will, however, need to be explored in future studies.

**Figure 8.**
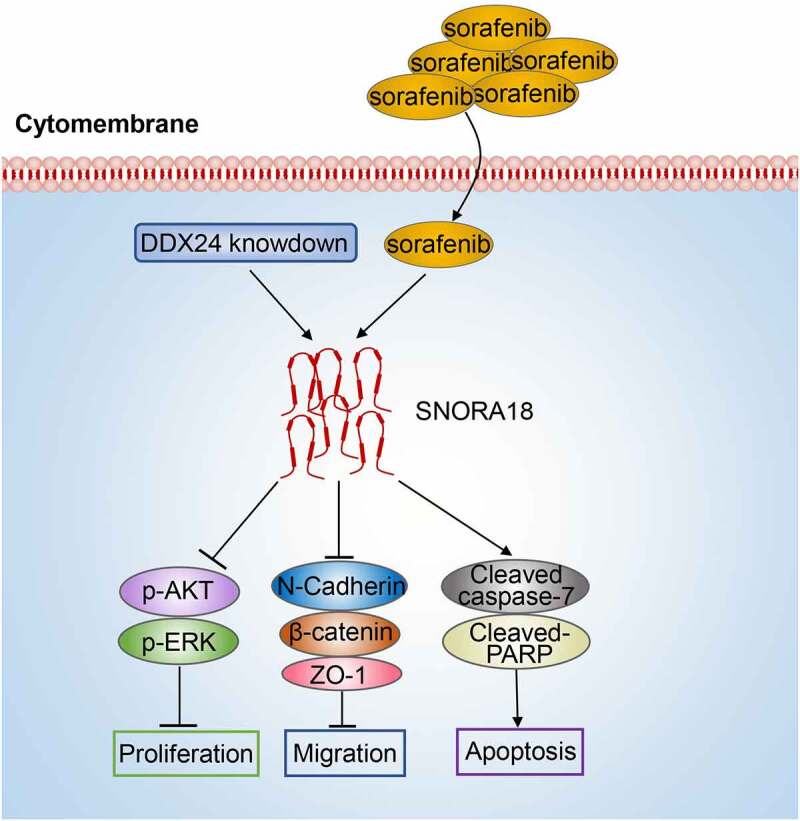
Schematic model: DDX24 modulates the chemosensitivity of SFN in HCC treatment via SNORA18 dependent pathway. DDX24 knockdown synergized with SFN enhanced the inhibitory effects of cell proliferation and migration via AKT/ERK and EMT pathway in HCC. Downregulation of DDX24 elevated sorafenib-induced apoptosis via caspase/PARP pathway in HCC. Mechanistically, DDX24 regulated SFN sensitivity in HCC treatment via mediating the expression of its target SNORA18.

In conclusion, by means of a bioinformatics analysis, the highlighted findings of our research identified DDX24 as an adverse indicator of HCC prognosis and that it was significantly related to the pathways modulating tumor development. Furthermore, we identified that DDX24 regulated the sensitivity of SFN in HCC treatment by mediating SNORA18 (Figure 8). Modulating the expression of DDX24 and its target SNORA18 may serve as effective and specific therapeutic strategies for HCC treatment in future.

## Materials and methods

### Cell culture

Hep3B human HCC cells were acquired from American Type Culture Collection (ATCC, Manassas, VA, USA), and Bel-7402 human HCC cells were obtained from Cell Bank of the Chinese Academy of Sciences (Shanghai, China). These cells were incubated in Dulbecco’s Modified Eagle Medium (DMEM; GIBCO, Catalog #C11995500BT, NY, USA) supplemented with 10% fetal bovine serum (FBS; ExCell Bio, Catalog #FSP500, Shanghai, China) and 0.1% penicillin-streptomycin in a humidified atmosphere containing 5% CO2 at 37 °C. Sorafenib (SFN; Selleck, Catalog #S7397, Texas, USA) was dissolved in DMSO and diluted at the indicated concentration before it was added to the complete medium for treatment. The cells were authenticated by short tandem repeat (STR) profiling and were confirmed to be mycoplasma free before use.

### Cell transfection

DDX24 shRNAs (shDDX24-1, 5’-GCUGCUAGAGAUGCUCAAUTT-3’; shDDX24-2, 5’-CCGUUUAGCUCGACAGAUUTT-3’), SNORA18 antisense oligonucleotide (SNORA18 ASO, 5’-UUACUCTATGAGGCGUUUCC-3’) and their corresponding negative control were synthesized by GenePharma Company (Shanghai, China). GV658-DDX24, GV658-SNORA18 and control vector plasmids were synthesized by Genechem Company (Shanghai, China). Transfection was performed with Lipofectamine 3000 (Invitrogen, Catalog #L3000015, California, USA) when cells in a 6-well plate had grown to the appropriate density (80%-90%) according to the manufacturer’s instruction. The cells were harvested for subsequent experiments after 48–72 hr of transfection.

### Generation of lentiviral particles and cell transduction

To establish HCC cell lines with DDX24 stable knockdown, Hep3B and Bel-7402 cells were infected with DDX24 knockdown lentivirus or empty vector (U6-sh-DDX24-EGFP-IRES-puromycin; GeneCopoeia, Rockville, Maryland, USA) according to the manufacturer’s instruction. After 72 hr, cells were grown in a medium containing 1.0 μg/ml of puromycin (Invitrogen, Catalog #P8230, California, USA) for antibiotic selection. The efficiency of RNA interference was evaluated by quantitative real-time PCR (RT-qPCR) and western blot analysis.

### Quantitative real-time PCR

Total RNAs were extracted using E.Z.N.A.® Total RNA Kit I (OMEGA, Catalog #R6834-02, Georgia, USA) or TRIzol reagent (Invitrogen, Catalog #abs60154, California, USA), and subsequently converted to cDNA using HiScript II One Step RT-PCR Kit (Vazyme, Catalog #R323-01, Nanjing, China) according to the manufacturer’s protocol. qPCR was employed to detect the expression of genes using All-in-OneTM qPCR Mix (GeneCopoeia^TM^, Catalog #P222-02, Rockville, Maryland, USA) on Bio-Rad CFX96 following the manufacturer’s instruction, and analysis using Bio-Rad Manager software (Bio-Rad, Hercules, CA). GAPDH or U6 was used as internal controls, and the 2^−∆∆Ct^ method was used to calculate the relative expression of genes. Primers for DDX24 (catalog no. Hs-QRP-23370), GAPDH (catalog no. Hs-QRP-20169), C15orf38-AP3S2 (catalog no. HQP055868), SLC6A6 (catalog no. HQP109841), RPL12P38 (catalog no. CS-QP0062) and SNORA18 (catalog no. CS-HmiR0023) were purchased from GeneCopoeia Inc (GeneCopoeia^TM^, Rockville, Maryland, USA). The sequence of U6 primer was forward: 5’-CGCTTCGGCAGCACATATAC-3’ and reverse: 5’-AAATATGGAACGCTTCACGA-3’.

### Western blot analysis

Whole cells were lysated in a RIPA lysis buffer (Beyotime Biotechnology, Catalog #P0013C, Shanghai, China) containing a protease/phosphatase Inhibitor Cocktail. BCA assays (Beyotime Biotechnology, Catalog #P0010, Shanghai, China) were used to detect the concentration of proteins in each sample. Proteins were separated by 5–20% SDS-PAGE gels (Genescript, Catalog #M42012C, Nanjing, China) and transferred onto PVDF membranes (Roche, Catalog #03010040001, Basel, Switzerland). After blocking with 5% milk for 1 hr at room temperature, primary antibodies were incubated overnight at 4 °C. After washing 3 times with TBST, a HRP-conjugated anti-rabbit or anti-mouse secondary antibody was incubated for 1 hr at room temperature. The protein bands were visualized with the use of a chemiluminescence kit (Thermo, Catalog #34580, Carlsbad, USA). The antibodies used are listed in Supplementary Table 2.

### Cell viability assay

Cell Counting Kit-8 (CCK-8; KeyGEN BioTECH, Catalog #KGA317, Nanjing, China) was used to assess cell viability. In brief, cells were plated in a 96-well plate (Corning, Catalog #3599, NY, USA) at a density of 3 × 10^3^ cells per well (48 hr after transfection) and incubated overnight at 37 °C. Cells were then treated with DMSO or indicated concentrations of SFN for 48 hr. Subsequently, the medium was removed and replaced with 100 μl 10% CCK-8 solution. The absorbance was detected at 450 nm using a microplate reader. The concentration of SFN causing 50% inhibition of HCC cell activity was defined as IC50. All experiments were repeated independently three times.

### Colony formation assay

Briefly, cells were plated in a 6-well plate (Corning, Catalog #3516-50, NY, USA) at a density of 2 × 10^5^ cells per well (48 hr after transfection) and incubated overnight at 37 °C. Then cells were treated with DMSO or SFN medium for 14 days, and the medium was changed once every three days. At the end of incubation, colonies were fixed with ethanol for 30 min and stained with 5% crystal violet (Beyotime Biotechnology, Catalog #C0121, Shanghai, China) for 1 hr. The number of colonies with >10 cells was calculated using ImageJ software. Each experiment was conducted at least three times.

### Cell apoptosis analysis

To detect the percentage of cell apoptosis, flow cytometry assays were used. Briefly, HCC cells were seeded into a 6-well plate at a density of 2 × 10^5^ cells (48 hr after transfection). Cells were then treated with indicated concentrations of SFN for 48 hr. Next, cells were resuspended in a binding buffer containing Annexin V-FITC and propidium iodide (PI) (KeyGEN, Catalog #KGA108, Nanjing, China) in accordance with the manufacturer’s guideline, after washing with PBS three times. Finally, apoptotic cells were detected by flow cytometry and analyzed through FlowJo 7.6 software. Each experiment was conducted at least three times.

### Cell migration assay

The ability of cell migration was detected by trans-well assays. Briefly, HCC cells (8x10^4^ cells per well, 48 hr after transfection) suspended in serum-free medium were placed into the upper chambers of a trans-well filter (Corning, Catalog #353097, NY, USA), while the lower chambers were supplemented with DMEM containing 10% FBS, placed in a 24-well plate (NEST, Catalog #702001, Jiangsu, China). Indicated concentrations of SFN were added to the medium both in the upper and lower chambers. After 24 hr, the migration cells located on the lower membrane were immersed with methanol for 30 min and stained with 5% crystal violet for 1 hr. Migration cells from three fields were selected randomly under a bright field microscope and counted using ImageJ software. All experiments were repeated independently three times.

### F-actin staining

Briefly, HCC cells were seeded into chamber slides (NEST, Catalog #801002, Jiangsu, China) at a density of 1 × 10^3^ cells (48 hr after transfection) and cultured for 24 hr. The cells were then exposed to indicated concentrations of SFN for 48 hr. Next, the cells were fixed with 4% paraformaldehyde for 30 min at 4°C, permeabilized in 1% Triton X-100 (Solarbio Life Science, Catalog #T8200, Beijing, China) for 30 min at room temperature, and incubated in a blocking buffer for 1 hr at room temperature. This was followed by staining with Phalloidin-iFluor 555 in the dark for 1 hr at room temperature so as to bind them to F-actin (1:50 dilution, Abcam, Catalog #ab176756, Cambridge, UK). They were washed again three times in PBST. Finally, Fluoroshield Mounting Medium with DAPI (Abcam, Catalog #ab104139, Cambridge, UK) was applied to the chamber slides and incubated for 20 min in the dark. The images of samples were pictured under a confocal microscope.

### Immunohistochemical staining

Tumor tissues excised from mice were fixed overnight in 10% neutral buffered formalin, dehydrated at a gradient concentration, and embedded in paraffin. The tissues were cut into 4 μm thick and fixed on the silicified glass slide. Subsequently, the IHC was carried out using a streptavidin–peroxidase-conjugated method. Briefly, the slides were deparaffinized, rehydrated, immersed in antigen retrieval solution, boiled at 100°C for 10 min, and incubated with a peroxidase inhibitor for 10 min at room temperature. Next, nonspecific binding was blocked with normal goat serum at room temperature for 1 hr, and incubated overnight at 4°C with primary antibodies. After secondary antibodies were incubated at room temperature for 1 hr, 3,3’-Diaminobenzidine tetrahydrochloride (DAB; ZSGB-BIO, Catalog #ZLI-9017, Beijing, China) and Mayer’ Hematoxylin solution (Solarbio Life Science, Catalog #G1080, Beijing, China) were followed. Microscopic images were obtained under a bright field microscope, and the protein expression was visualized by IHC staining and evaluated using the CellProfiler software. The information relating to the antibodies are summarized in Supplementary Table 2.

### RNA-sequencing analysis

Total RNAs were extracted using the TRIzol reagent. Then strand-specific RNA-seq libraries were constructed using each group of samples and sequenced using a Illumina NovaSeq 6000 RNA-Seq System (Illumina, USA). Read counts for each gene were normalized into Fragments Per Kilobase of transcript per Million mapped reads (FPKM) values. Differentially expressed genes (DEGs) with log_2_FC ≥1.5 or ≤-1.5 fold change, *p < .01* were identified, and normalized using respective negative and vector controls. Differentially expressed genes (log_2_FC ≥1.5, *p < .01*) from the results of RNA-seq on Bel-7402 cells administrated with SFN and DDX24 knockdown Hep3B cells (GEO Submission: GSE145635) were included in the venn diagram, and the diagram of simultaneous upregulated gene lists were created using a free online tool (http://bioinformatics.psb.ugent.be/webtools/Venn/).

### Animal experiment

Animals were purchased from the Vital River Laboratory Animal Technology Company (Beijing, China), and maintained in a pathogen-free condition in Guangdong Provincial Key Laboratory of Biomedical Imaging under standard conditions at the animal care facility. BALB/c female nude mice aged four to six weeks were randomly divided into four groups (n = 5): shNC+PBS group, shNC+SFN group, shDDX24-1+ SFN group and shDDX24-2+ SFN group. Hep3B cells (5x10^6^ per mouse) with or without DDX24 stable knockdown were suspended in 100 μl PBS and injected subcutaneously into the right flanks of the animals. After five days, the mice were treated with SFN (60 mg/kg body weight) or parallel PBS as a control, orally once daily by gastric infusion, and the length, width, and height of each tumor were monitored every four days until the end of this study (tumor volume was calculated as follows: V = πLWH/6). Finally, the mice were humanely sacrificed by anesthesia, and xenografts were harvested for weighing and photographing. The relative expression of proteins in tumor tissues was detected by IHC assays. Animal protocols were approved by the Institutional Animal Care and Use Committee of the Fifth Affiliated Hospital of Sun Yat-sen University (No. 00087). All animals procedures were performed in accordance with the principles of the Declaration of Helsinki.

### Data acquisition, Kaplan-Meier survival and pathway enrichment analysis

Gene expression and clinical data for 371 liver cancer samples and 50 adjacent samples were obtained from The Cancer Genome Atlas (TCGA) database of UCSC Xena (https://xenabrowser.net/datapages/). This dataset showed the gene-level transcription estimates, as in log_2_(x + 1), and transformed the RSEM normalized count.

Kaplan-Meier analysis was conducted for the high and low DDX24 expression groups based on the median mRNA level of DDX24 in the TCGA cohort to assess the ability to predict patient survival.

The relationships between DDX24 and its neighboring genes were investigated via the GeneMANIA tool (http://genemania.org/), then these neighboring genes were used to construct a gene network map. The protein-protein interaction (PPI) network for DDX24 was constructed via the STRING database (https://string-db.org/cgi/). Co-expressed genes of DDX24 (*p < .05*) were screened from the TCGA database. The interaction network analysis of the top 50 co-expressed genes was analyzed via the Reactome Pathway database (https://www.reactome.org/), and the results of 34 interacting genes were visualized using Cytoscape tool (V 3.7.2). Then the top 18 co-expressed genes (|Spearman correlation coefficient| >0.4 and *p < .01*) were integrated into the Kyoto Encyclopedia of Genes and Genomes (KEGG) pathway analysis (https://david-d.ncifcrf.gov/). The results of the KEGG analysis were visualized using the R package (V 3. 6. 2).

### Statistical analysis

All data were presented in terms of the mean ± SD from at least three independent experiments. Statistical analysis was performed using GraphPad Prism software (version 7.0). The differences between groups were analyzed using two student’s *t* test, or by one-way ANOVA. Statistical significance was indicated as follows: ****p < .0001; ***p < .001; **p < .01; *p < .05; n. s. represented not significant.

## Supplementary Material

Supplemental Material

## Data Availability

The data used to support the findings of this study are included within the article. The data and materials in the current study are available from the corresponding author on reasonable request.
