# Supplementary material for: DDX24 regulates the chemosensitivity of hepatocellular carcinoma to sorafenib via mediating the expression of SNORA18
Source: Cancer Biol Ther. 2022 Oct 30;23(1):1–14. doi: 10.1080/15384047.2022.2135960 (PMC9629112; doi:10.1080/15384047.2022.2135960)
Supplement: Supplemental Material [file KCBT_A_2135960_SM6285.docx]

**Supplementary Table 1. Top 18 co-expressed genes of DDX24 in liver cancer samples**

| Gene | Spearman | *p* value |
| --- | --- | --- |
| DDX24 | 1 | <0.01 |
| ALKBH1 | 0.534150037 | <0.01 |
| PPP2R5C | 0.473187796 | <0.01 |
| GPHN | 0.47167733 | <0.01 |
| EIF2S1 | 0.459565069 | <0.01 |
| TSC22D1 | 0.458654722 | <0.01 |
| BAG5 | 0.440958078 | <0.01 |
| SETD3 | 0.435595944 | <0.01 |
| SNW1 | 0.433202715 | <0.01 |
| COQ6 | 0.426040004 | <0.01 |
| IFNGR1 | 0.424442514 | <0.01 |
| WDR20 | 0.417841246 | <0.01 |
| ZNF410 | 0.415125806 | <0.01 |
| ZFYVE21 | 0.415109325 | <0.01 |
| TECPR2 | 0.412891371 | <0.01 |
| SH3BP5 | 0.410548654 | <0.01 |
| KLC1 | 0.40713963 | <0.01 |
| GOLGA5 | 0.401875617 | <0.01 |
| RRAGA | 0.400307679 | <0.01 |

**Supplementary Table 2. The characteristics of antibodies**

| **Protein** | **Application** | **Origin** | **City/State/Country** | **Dilution** |
| --- | --- | --- | --- | --- |
| DDX24 | WB | A300-697A, Bethyl Laboratories | Montgomery/TX/USA | 1:1000 |
| β-actin | WB | #4970S, Cell Signaling Technology | Danvers/MA/USA | 1:1000 |
| GAPDH | WB | #51332, Cell Signaling Technology | Danvers/MA/USA | 1:1000 |
| AKT | WB | #4685S, Cell Signaling Technology | Danvers/MA/USA | 1:1000 |
| p-AKT | WB | #13038S, Cell Signaling Technology | Danvers/MA/USA | 1:1000 |
| ERK | WB | #4695S, Cell Signaling Technology | Danvers/MA/USA | 1:1000 |
| p-ERK | WB, IHC | #4370S, Cell Signaling Technology | Danvers/MA/USA | 1:1000 & 1:200 |
| ZO-1 | WB, IHC | #13663S, Cell Signaling Technology | Danvers/MA/USA | 1:1000 & 1:200 |
| N-cadherin | WB | #13116S, Cell Signaling Technology | Danvers/MA/USA | 1:1000 |
| β-Catenin | WB, IHC | #8480S, Cell Signaling Technology | Danvers/MA/USA | 1:1000 & 1:200 |
| PARP | WB | #9542S, Cell Signaling Technology | Danvers/MA/USA | 1:1000 |
| Cleaved caspase-7 | WB | #8438S, Cell Signaling Technology | Danvers/MA/USA | 1:1000 |
| Ki67 | IHC | bs-23105R, Bioss | Beijing/China | 1:200 |
| HRP-conjugated anti-rabbit | WB | 310802, EarthOx Life Sciences | Millbrae/California/USA | 1:5000 |
| HRP-conjugated anti-mouse | WB | 920702, EarthOx Life Sciences | Millbrae/California/USA | 1:5000 |
